# Blocking BAFF Alleviates Hepatic Fibrosis in *Schistosoma japonicum*-Infected Mice

**DOI:** 10.3390/pathogens12060793

**Published:** 2023-06-01

**Authors:** Panpan Dong, Congjin Mei, Yingying Yang, Yonghua Zhou, Yongliang Xu, Lijun Song, Chuanxin Yu

**Affiliations:** National Health Commission Key Laboratory of Parasitic Disease Control and Prevention, Jiangsu Provincial Key Laboratory on Parasite and Vector Control Technology, Jiangsu Provincial Medical Key Laboratory, Jiangsu Institute of Parasitic Diseases, Wuxi 214064, China

**Keywords:** *Schistosoma japonicum*, B-cell-activating factor, liver fibrosis, anti-BAFF treatment, apoptosis

## Abstract

Schistosomiasis is an immunopathogenic disease characterized by egg granuloma and fibrosis. The hepatic fibrosis of schistosomiasis is caused by the coordinated action of local immune cells, liver-resident cells and related cytokines around the eggs of the liver. B-cell-activating factor (BAFF), expressed in many cells, is an essential factor for promoting the survival, differentiation, and maturation of cells. The overexpression of BAFF is closely related to many autoimmune diseases and fibrosis, but has not been reported to play a role in liver fibrosis caused by schistosomiasis. In the study, we found that, during *Schistosoma japonicum* (*S. japonicum*) infection in mice, the level of BAFF and its receptor BAFF-R progressively increased, then decreased with the extension of infection time, which was consistent with the progression of hepatic granuloma and fibrosis. Anti-BAFF treatment attenuated the histopathological damage in the liver of infected mice. The average areas of individual granulomas and liver fibrosis in anti-BAFF treatment mice were significantly lower than those in control mice, respectively. Anti-BAFF treatment increased the IL-10, decreased IL-4, IL-6, IL-17A, TGF-β, and downregulated the antibody level against *S. japonicum* antigens. These results suggested that BAFF acts a strong player in the immunopathology of schistosomiasis. Anti-BAFF treatment may influence Th2 and Th17 responses, and reduce the inflammatory reaction and fibrosis of schistosomiasis liver egg granuloma. It is suggested that BAFF might be a prospective target for the development of new methods to treat schistosomiasis liver fibrosis.

## 1. Introduction

Schistosomiasis is a disease that can cause a series of systemic immunopathogenic changes. One of the main characteristics is hepatosplenomegaly [1]. The inflammatory reaction of egg granuloma and the ensuing fibrosis are the pathological bases of cirrhosis caused by schistosomiasis [2]. Although many candidate agents, including praziquantel, have showed effective anti-fibrotic effects in animal models or limited clinical trials, there are no approved therapeutic drugs at present [3,4].Therefore, it is necessary to further study the molecular mechanism of schistosomiasis hepatic fibrosis in order to set the groundwork for the progression of novel anti-schistosomiasis liver fibrosis methods.

There is evidence indicating interactions in the immune system, especially the interplay between T cell, B cells, and fibroblasts, leading to fibrosis [5]. The early host immune response of mice infected with *S. japonicum* was mainly a Th1 type immune response, and the IFN-γ and IL-12 secreted by CD4^+^ T cells played an important role in promoting inflammatory response. With the infection time, the host immune response shifted to Th2 type, producing IL-4, IL-5, IL-10, IL-13, and TGF-β, and promoting the formation of fibrosis [6]. In mice infected with *S. japonicum*, B cell response appeared 5–6 weeks after infection, peaking at 8 weeks and then declined, which was related to the hepatic pathological changes induced by *S. japonicum* [7]. At the early stage of infection, the accumulation of B cells and the antibodies produced in the granuloma indicate that the B cell response plays an important role in the inflammatory response of granuloma [8]. In recent years, B cell subsets and their related factors were found to play a dual regulatory role in schistosome infection [9]. B cells could be divided into B-1 cells, B-2 cells, effector B cells (Beffs) and regulatory B cells (Bregs) according to the differences in their surface molecules, localization and functional characteristics. The secretion of IL-10 by hepatic B-1 cells was significantly increased after schistosome infection, and IL-10 attenuated inflammation and liver fibrosis by decreasing the expression of inflammatory factors and chemokines and inhibiting the infiltration of Ly6c^hi^ monocytes in the liver [10]. Beffs produces IL-6, promoting a Th2 immune response [11]. Beffs not only plays a role in adaptive immunity through antibodies, but also plays a role in parasitic infections through the production of cytokines [12]. *S. japonicum* infection regulates the differentiation and development of B cell subsets, and B cells influence the response of CD4^+^ T cells through the secretion of TGF-β and expression of surface-programmed death protein ligand 1 [9]. Overactivity of the CD4^+^ effector T cells and the overproduction of proinflammatory cytokines contributes to the development of immune-mediated inflammation and multi-organ fibrosis [13].

B-cell-activating factors (BAFF) is a member of the tumor necrosis factor superfamily and participate in the immune response of many diseases by regulating the functions of T and B cells. BAFF is also known as BLys, THANK, TALL-1, zTNF4, TNFSF-13B and CD257. It is mainly expressed in the cells of the marrow system, as well as other cells such as epithelial cells [14]. BAFF receptor (BAFF-R) is one of its specific binding receptors, expressed not only on B cells but also Tregs, and is increased in activated T cells [15]. After binding with BAFF-R, BAFF contributes to cell survival, maturation, proliferation and differentiation, participates in the conversion of antibody categories, and co-stimulates T cell activation [16]. BAFF also has an important immunomodulatory function, improving the expression of CD4^+^Foxp3^+^ Tregs in the spleen and tumor microenvironment [17]. Abnormal BAFF signaling could cause immune imbalances and is tightly correlated with the occurrence and development of a variety of autoimmune diseases, such as systemic lupus erythematosus (SLE) [18,19], rheumatoid arthritis [20], and multiple sclerosis (MS) [21]. Recently, B cells have been shown to be implicated in the process of tissue fibrosis in several diseases, including transplant immunity, autoimmune diseases, liver fibrosis, and pulmonary fibrosis [22]. BAFF may play a differential diagnostic role in idiopathic pulmonary fibrosis (IPF), which is associated with autoimmune diseases (AIDs) [23]. Xu et al. first proved that BAFF signal was directly related to renal fibrosis [24]. Improvements in BAFF levels were related to the severity of the disease, while reduced BAFF levels were in line with a reduction in skin fibrosis [25]. Pre-fibrosis cytokines and direct cell–cell contact may participate in the fibrosis mediated by TGF-β1 and induced by B cells and BAFF [25]. Studies have shown that BAFF blockades could attenuate fibrosis. Blocking BAFF reshaped the hepatic B cell receptor repertoire and attenuated the production of autoantibodies in cholestatic liver disease, alleviating liver fibrosis in wild-type mice. BAFF depletion improved the insulin resistance and hepatic steatosis induced by a high-fat diet (HFD), alleviating inflammation and fibrosis in a murine model of nonalcoholic fatty liver disease [26,27]. Using anti-BAFF in autoimmune diseases could inhibit B cell maturation and survival, which reduced the tissue fibrosis level [22,28]. Therefore, BAFF may become a new therapeutic target for fibrotic lesions. However, the effect of BAFF on the hepatic fibrosis caused by *S. japonicum* remains unclear.

In this study, the relationship between the dynamic changes in BAFF and the pathological process during *S. japonicum* infection were investigated, and the effect of anti-BAFF treatment on liver fibrosis in *S. japonicum* infected mice was observed. The results showed that the serum BAFF level in mice was related to the liver progression of *S. japonicum* infection, and anti-BAFF treatment could effectively reduce liver fibrosis. These findings indicated that BAFF was important to the pathogenesy of schistosomiasis japonicum, which helped to further clarify the mechanism of schistosomiasis liver fibrosis and develop new anti-fibrosis measures.

## 2. Materials and Methods

### 2.1. Schistosome and Animals

The infected *Oncomelania* snails were provided by Jiangsu Institute of Parasitic Diseases. The incandescence of infected *Oncomelania hupeensis* occurred in dechlorinated water at 20–25 °C for 2 h (hours) to incubate cercariae.

Specific pathogen-free (SPF) grade 6-week-old C57BL/6J mice (female, 22 ± 2 g) were bought from the Yangzhou University Comparative Medical Center. All experimental animals were kept in the Jiangsu Institute of Parasitic Diseases Experimental Animal Center and had free intake of food and water under a 12 h diurnal cycle.

### 2.2. Mice Infected with S. japonicum

To test whether BAFF levels in mice infected with *S. japonicum* were associated with disease progression, mice were randomly divided into four groups. Each group contained six mice. Each mouse in the infection groups was infected with 15 ± 2 *S. japonicum* cercariae via the abdominal skin. Mice were sacrificed at 0, 3, 6, 9 W (week) post-infection, separately. The adult worms were harvested by perfusion through the portal vein and mesenteric vein of infected mice. After weighing part of the right lobe of the liver, it was digested with 10 mL 4% potassium hydroxide (KOH) at 37 °C overnight and the eggs were counted to determine the egg burden of per gram of livers. Mice, spleens and livers were weighed, and spleen/liver index was calculated. Spleen index was expressed as spleen weight (mg)/body weight (g). Liver index was expressed as liver weight (mg)/body weight (g) [29]. At 0, 3, 6, 9 W post-infection, mouse blood was gained from the submandibular vein plexus. The serum was isolated from the blood and stored at −80 °C. Splenocyte was isolated by physical fragmentation and examined by flow cytometry.

### 2.3. S. japonicum Infected Mice Treated with Anti-BAFF Antibody

To investigate the role of BAFF in promoting liver fibrosis of schistosomiasis, mice were randomized into four groups: control group (control, no treatment), infection group infected with *S. japonicum* (Sj, each mouse infected with *S. japonicum*), istoype control treated group infected with *S. japonicum* and with treatment of mouse IgG1 isotype control (Sj + IgG1 control), anti-BAFF-treated group infected with *S. japonicum* and with treatment of monoclonal antibody anti-BAFF (Sj + anti-BAFF). Sj + IgG1 control group were treated i.p. at week 3 after infection (single administration) with mouse IgG1 isotype control (15H6, IgG1; AdipoGen, SanDiego, CA) (at 2 mg/kg) by further injections every 2 weeks. Sj + anti-BAFF group were treated i.p. at week 3 after infection with monoclonal antibody anti-BAFF (mouse), mAb (Sandy-2) (single dose) (preservative-free) (single dose) (SANDY-2; AdipoGen, SanDiego, CA) (at 2 mg/kg) by further injections of Sandy-2 every 2 weeks. Schistosomes are paired at 3 weeks and lay eggs about 24 days post infection. Therefore, we chose to administer antibody neutralization therapy three weeks after infection to regulate the host’s immune function and observe whether it can alleviate the subsequent inflammatory response and fibrotic lesions induced by antigens released from the egg. The usage and dosage of antibody were determined according to the instructions and previous literature reports [30]. Each group contained six mice. The serum was also collected and stored as above. The number of worms and eggs were counted as the above procedure. Mice and spleens, livers were weighed and spleen index was calculated to determine the spleen/liver index as above.

### 2.4. Determination Hydroxyproline Content in the Liver of Mice

The liver (30–100 mg) was digested with a hydroxyproline detection kit (Nanjing Jiancheng Bioengineering Research Institute Co., Ltd., Nanjing, China), and hydroxyproline detection was performed according to the manufacturer’s protocol. Microplate reader (model 570, Bio-Rad, Hercules, CA, USA) was used to measure the absorbance at 550 nm (A550). The amount of hydroxyproline was expressed as micrograms per gram of liver relative to the standard curve.

### 2.5. HE and Masson Collagen Staining

Right anterior liver sections were fixed overnight in 4% paraformaldehyde, dehydrated, paraffin-embedded, cut into 4 μm sections and processed with hematoxylin/eosin (H&E) and Masson trichrome staining (Nanjing Jiancheng Biotechnology, Nanjing, China). Granulomas containing individual egg were photographed using an Olympus BX51 microscope (Olympus Co., Tokyo, Japan) and the areas of granulomas were measured using Cellsens Dimension software (Olympus Co., Tokyo, Japan). From three to five sections were counted per mouse, 20 or more granulomas were measured, and the mean area was calculated [31].

### 2.6. Enzyme-Linked Immunosorbent Assay (ELISA)

BAFF levels in serum were determined with Mouse BAFF/BLys/TNFS13B Immunoassay Kit (R&D Systems) after the intervention was completed, following the kit instructions [26]. IL-4, IL-6, IL-17A, IFN-γ, and TNF-α in serum were determined with Mouse High-Sensitivity ELISA kits (MULTI SCIENCES (LIANKE) BIOTECH, CO., LTD., Hangzhou, China). TGF-β1 was assayed by TGF-β1 ELISA kit (MULTI SCIENCES). IL-10 levels were detected with a Mouse IL-10 ELISA kit (MULTI SCIENCES). ELISAs were developed according to the kit instructions, and read with a microplate reader (model 570, Bio-Rad, USA).

Specific IgG antibodies against *S. japonicum* adult worm antigen (AWA) and soluble egg antigen (SEA) in mice serum were determined by ELISA. SEA and AWA of *S. japonicum* were prepared by our laboratory, as has been noted [32]. In brief, antigens were diluted with coating buffer to a final concentration of 10 μg/mL; 100 μL of antigen was added to each well and incubated overnight at 4 °C. Firstly, each well was blocked with blocking regent (1% BSA in PBST) for 1 h at 37 °C. Secondly, the serum samples were diluted 100-fold with blocking buffer, added to each well (100 μL/well) and incubated for 1 h at 37 °C. HRP-sheep anti-mouse IgG (Bethyl, Montgomery, TX, USA) was used as the secondary antibody (1:20,000, 100 μL/well), and the samples were incubated for 1 h at 37 °C. The wells were washed five times with PBST. The results were developed with TMB substrate (50 µL/well) for 5 min and then stopped with 2 M sulphuric acid solution (50 µL/well). Record optical density (OD) values were measured at 450 nm using a microplate reader. Samples were analysed in duplicate [33].

### 2.7. Flow Cytometry

The physical grinding method was used to isolate spleen cells. The expression levels of BAFF and BAFF-R on splenocytes after *S. japonicum* infection were determined by flow cytometry (BD FACSVerse™). Cells were treated with anti-CD16/32 (2.4G2) (BD Bioscience, San Jose, CA, USA) to block FcγR II/III. FITC-anti-mouse CD19, PE-anti-mouse BAFF-R and APC-anti-mouse BAFF-conjugated antibodies were then used for cytokine staining on the cell surface. Cell staining was analysed using FACS Suite (BD Bioscience) or FCS Express (De Novo Software, Los Angeles, CA, USA).

### 2.8. Statistical Analysis

Measurement data were expressed as mean ± SEM, and two independent samples means were compared by *t* or Mann–Whitney *U* tests. ANOVA was used to compare 3 or more groups. A *p*-value of < 0.05 was considered to be significant.

## 3. Results

### 3.1. The Dynamic Changes in BAFF, BAFF-R Were Related to the Pathological Course of Schistosomiasis

**Figure 1:**
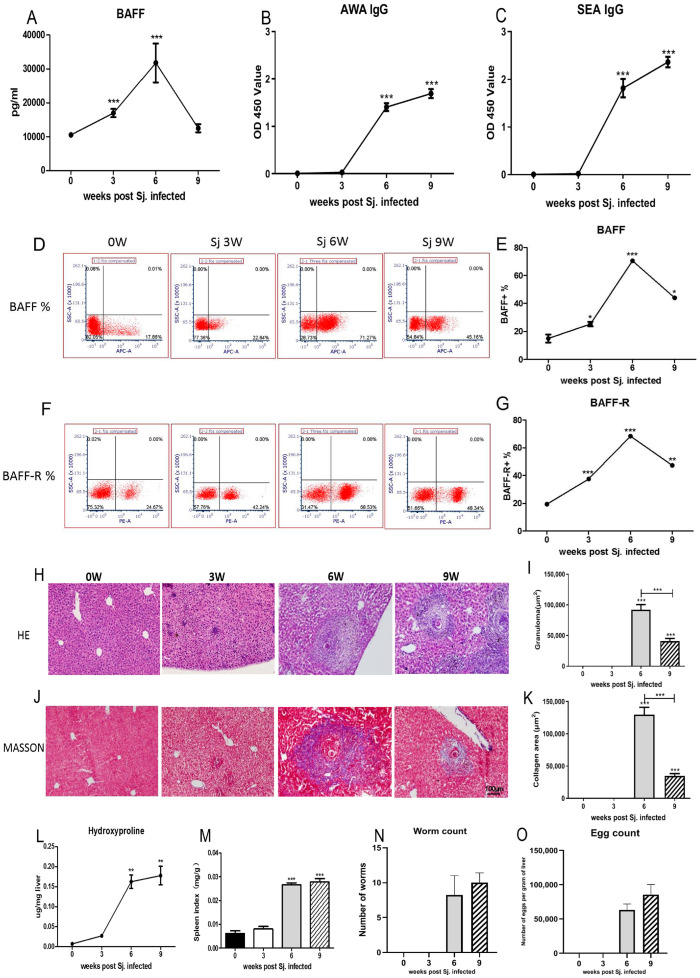
The BAFF and BAFF-R levels were correlated with the progress of schistosomiasis japonicum. C57BL/6 mice were infected with 15 ± 2 *S. japonicum* cercariae via the abdominal skin. At 0, 3, 6 and 9 weeks following infection, sera, liver and spleen were collected for experimentation. (**A**) BAFF levels in serum. The levels of serum specific IgG antibody against the AWA (**B**) and SEA (**C**) were measured by ELISA. Cells in spleen were harvested and processed for FACS staining. Cells were stained for BAFF (**D**) and BAFF−R (**F**). BAFF^+^ cells (**E**) and BAFF−R^+^ cells (**G**) were summarized in line graphs. Hematoxylin and Eosin (H&E) staining (×100) (**H**) and Masson’s trichrome (**J**) staining (×100) are shown in liver slices. Masson trichrome staining was used to measure the size of granulomas (**I**) and collagen areas (**K**) around a single egg. The level of hydroxyproline in the liver (**L**) was determined. (**M**) Spleen index was expressed as spleen weight (mg)/body weight (g). (**N**) Adult worms’ burdens. (**O**) Eggs’ burdens. The mean and SEM values were calculated. * *p* < 0.05, ** *p* < 0.01, *** *p* < 0.001 vs. 0 W group.

Mice sera at 0, 3, 6, 9 W were collected to investigate the change in BAFF level, as well as in specific antibody IgG against SEA and AWA during disease progression after infection with *S*. *japonicum*. The serum BAFF of mice increased rapidly and peaked at week 6, and then declined post infection (Figure 1A). The anti-AWA and anti-SEA specific IgG levels in serum increased gradually with the duration of infection (Figure 1B,C). The expression of BAFF (Figure 1D) and BAFF-R (Figure 1F) in spleen cells also peaked at week 6, and then declined (Figure 1E,G). A granuloma size of approximately a single egg was measured in H&E-stained liver sections (Figure 1H). The sizes of the granuloma increased with the prolongation of infection time, peaked at 6 weeks, and then declined (Figure 1I). The fibrotic areas surrounding granulomas were confirmed by Masson’s trichrome staining (Figure 1J). The sizes of the fibrotic areas also increased with the prolongation of infection time, peaked at 6 weeks, and then declined (Figure 1K). Hepatic hydroxyproline (an amino acid that is specific to fibrillar collagens, making up approximately 13.5% of the protein) and the spleen index increased with the prolongation of infection time (Figure 1L, M). The average number of worms showed no significant differences between the 6 W and 9 W group (Figure 1N). The 9 W group had a higher number of eggs than the 6 W group, but the average number of eggs showed no significant differences between the 6 W and 9 W group. At 0 W and 3 W, the worms and eggs were not detectable (Figure 1O). These results showed that the dynamics of BAFF and BAFF-R correlated with the pathologic course of schistosomiasis.

### 3.2. Anti-BAFF Treatment Inhibited Hepatic Fibrosis of Mice Infected with S. japonicum

In order to further validate the role of BAFF in promoting hepatic fibrosis, *S. japonicum*-infected mice were treated with anti-BAFF mAb (Sandy-2) neutralizing BAFF. Sandy-2 or isotype control mAb was injected intraperitoneally into the animals at 2 mg/kg. Mice were sacrificed at 7 W post infection.

**Figure 2:**
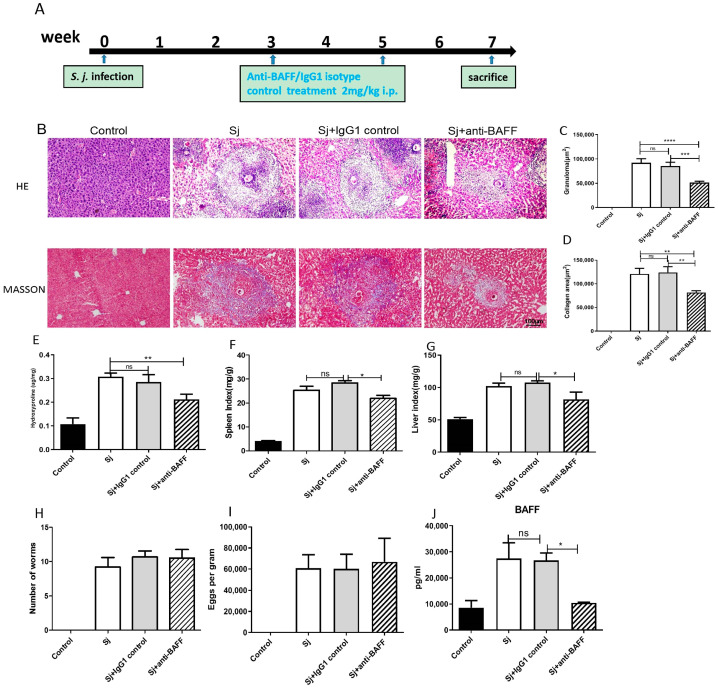
Anti-BAFF treatment suppressed the formation of liver granulomas and fibrosis in mice. C57BL/6 mice were infected with 15 ± 2 *S. japonicum* cercariae via the abdominal skin. At 7 weeks following infection, sera, liver and spleen were collected for experimentation. (**A**) Schematic diagram of the experimental design of anti-BAFF treatment for mice infected with *S. japonicum.* (**B**) The pathological changes were shown with representative hematoxylin and eosin (H&E) and Masson trichrome staining (×100). (**C**) Granuloma size and (**D**) collagen areas around a single egg were measured. (**E**) The level of hepatic hydroxyproline was determined. (**F**) The spleen index was expressed as weight of spleen (mg)/body weight (g). (**G**) Hepatic index was expressed as liver weight/body weight. (**H**) The average burden of adult worms per mouse of different group collected from portal and mesenteric vein. (**I**) The average egg burden per gram in liver tissue of different groups. (**J**) Serum BAFF level of mice in different groups. The mean values and the SEM values were calculated. ns = not significant, * *p* < 0.05, ** *p* < 0.01, *** *p* < 0.001, **** *p* < 0.0001.

HE staining exhibited that the control group had normal mouse liver structure, and the liver cells presented radial arrangement and normal morphology. After infection, the mouse liver was significantly granulomatous, surrounded by a large amount of inflammatory cell infiltration, nearby hepatocyte degeneration and necrosis, and the hepatic sinuses were significantly dilated and distorted (Figure 2A). The mean area of liver egg granuloma in the group infected with *S. japonicum* for 7 W (Sj) was markedly larger compared to that in the Sj + anti-BAFF group (*p* < 0.001, Figure 2B). Masson staining showed that the control group had a few collagen (Col) fibers in the portal area and central vein wall of the liver tissue. After infection, collagen fibers were deposited around the egg granuloma in the liver tissue. The amount of collagen fibers significantly increased with the extension of infection time, and extended to the hepatic lobules. Microscopically, eggs and peripheral egg granulomas were seen in the liver, and the collagen fiber content in the granulomas gradually increased with the progression of fibrosis (Figure 2A). The collagen area around eggs in the Sj group was significantly greater than that in the Sj + anti-BAFF group (*p* < 0.01, Figure 2C). Hydroxyproline content also significantly decreased in anti-BAFF-treated groups (*p* < 0.01, Figure 2D). The spleen index of the infected mice increased with the duration of infection, which, in the antagonist treatment group, significantly decreased (*p* < 0.05, Figure 2E). Liver index showed the same result (*p* < 0.05, Figure 2F). Determination of worm burden showed that the antagonism of BAFF had no effect on the *S. japonicum* worm burden (*p* > 0.05, Figure 2G) and fecundity (*p* > 0.05, Figure 2H). BAFF blockade could significantly decrease the BAFF level in serum. At 7 W after infection, the Sj group had considerably higher BAFF levels in serum than that of control group, while the Sj group had also a significantly higher level of BAFF than that of the Sj + anti-BAFF group (*p* < 0.05). The IgG1 isotype had no effects on BAFF level (*p* > 0.05, Figure 2I). These results showed that BAFF blockade could reduce the formation of liver granulomas and the subsequent fibrosis triggered by *S. japonicum* infection.

### 3.3. Anti-BAFF Treatment Decreased the Specific IgG against S. japonicum Antigens and Fibrosis-Related Cytokines Level in Serum

**Figure 3:**
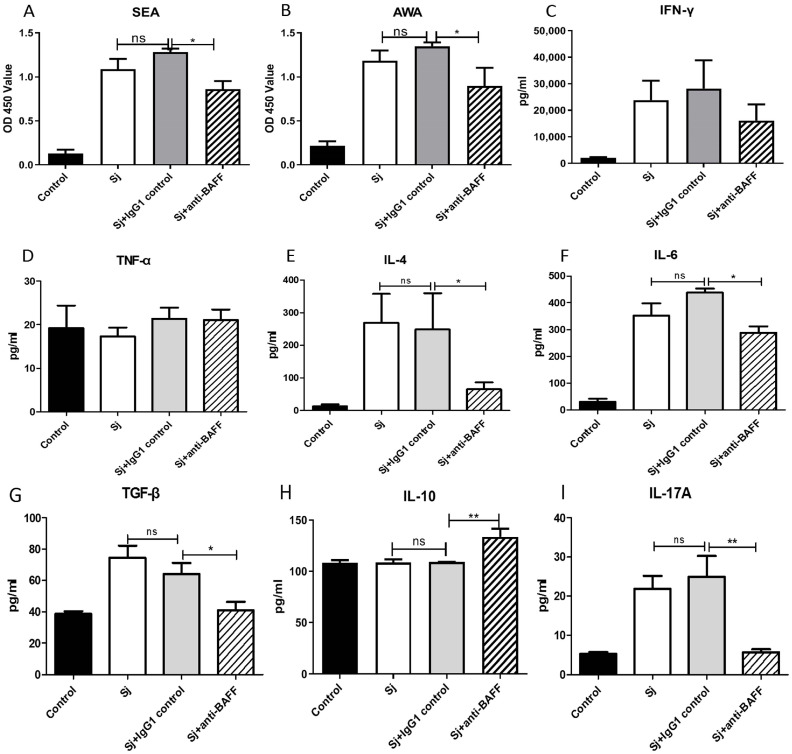
Effects of anti-BAFF treatment on the level of anti-SEA IgG, anti-AWA IgG and cytokines in serum. (**A**) Anti-SEA IgG levels, (**B**) Anti-AWA IgG levels, (**C**) IFN-γ levels, (**D**) TNF-α levels, (**E**) IL-4 levels, (**F**) IL-6 levels, (**G**) TGF-β levels, (**H**) IL-10 levels, (**I**) IL-17A levels were assayed. All experiments were performed in triplicate and the mean and SEM values were calculated. ns = not significant, * *p* < 0.05, ** *p* < 0.01,.

In order to examine the effect of BAFF in schistosomiasis, the impact of anti-BAFF treatment on the antibody-specific IgG aganst *S. japonicum* and the inflammation cytokines of mice infected with *S. japonicum* were observed. The findings indicated the following: anti-BAFF treatment could significantly decrease the anti-SEA- and anti-AWA-specific IgG levels in serum (*p* < 0.05, Figure 3A,B). Compared to the control group, the IFN-γ levels in mice increased after infection in other three groups, but no significant difference was observed among these three infection groups (Figure 3C). TNF-α level did not significantly change among different groups (Figure 3D). IL-4 (Figure 3E), IL-6 (Figure 3F), TGF-β (Figure 3G) and IL-17A (Figure 3I) levels in serum were markedly upregulated in mice infected with *S. japonicum*, and downregulated in mice of the Sj + anti-BAFF group. IL-10 levels increased after infection and markedly upregulated in mice of Sj + anti-BAFF group (*p* < 0.01, Figure 3H).

## 4. Discussion

Schistosomiasis japonicum is a widespread parasitic diseases, which seriously threatens people’s health in China [34,35]. Exploration of the immune mechanism of the egg granuloma inflammation would be helpful for developing new methods to reduce the harm caused by schistosomiasis. In the present work, we showed that BAFF and BAFF-R levels rapidly increase in mice after infection with *S. japonicum*, which is related to the disease progression. After *S. japonicum* infection, BAFF levels, hepatic granulomatous inflammation, and fibrosis degree both showed an initial increase, followed by a decrease. These results suggest that BAFF plays a major role in the granulomatous inflammation and fibrotic lesions induced by schistosome infection.

Previous studies have shown that the use of BAFF antagonist could alleviate skin fibrotic lesions [36]. Antagonizing BAFF not only inhibited the production of fibrogenic cytokines, but also increased the secretion of anti-fibrogenic cytokines, ultimately contributing to the attenuation of skin fibrosis [25]. Anti-BAFF and anti-CD20 antibodies may reduce the degree of tissue fibrosis in autoimmune diseases by depleting pre-B cells [22]. Therefore, the anti-BAFF monoclonal antibodies were used to verify the role of BAFF in egg granuloma inflammation and hepatic fibrosis. The results showed that blocking BAFF could reduce the degree of hepatic fibrosis caused by schistosomiasis, which agrees with previous studies. In this process, B cells are the main effector cells, and could differentiate into plasma cells after activation and assume an antibody secretion function [37]. Therefore, anti-SEA- and anti-AWA-specific IgG levels in serum were detected. Related studies have shown that a high level of anti-Schistosoma IgG is associated with increased susceptibility to parasites and that anti-Schistosoma IgG, especially the IgG4 response, is positively correlated with severe schistosomiasis [38]. Consistent with the expectations, BAFF blockade could significantly decrease the level of IgG against SEA and AWA, indicating that blocking BAFF could effectively inhibit B cell antibody secretion function. Since anti-BAFF treatment has not reduced the adult worm burden and eggs burden in infected mice, it is suggested that the reduction in pathological damage may be related to the reduction in inflammatory response induced by schistosomiasis antigen.

In addition to secreting antibodies, effector B cells (Beffs) are also able to secrete IL-2, IL-4, IL-6, IL-12, IL-13, IL-15, GM-CSF and IFN-γ, which are key cytokines regulating immune system’s assembly and function [39]. Yong et al. [10] showed that B1 cells in the liver played an important role in protecting against schistosomiasis liver disease by regulating IL-10 secretion. Regulatory B cells (Bregs) are a complex population that express IL-10, IL-35 and/or TGF-β and are capable of inhibiting Th1, Th17 and CD8^+^ responses; conversely, they could favor the development of regulatory T cells, impeding the resolution of the pathology [40]. Furthermore, BAFF could affect Th17 cell production and function, promoting CD4^+^ T cell differentiation by enhancing IFN-γ^+^ cell and IL-17^+^ T cell differentiation and inhibiting IL-4^+^ T cells [41].

T- and B-cell-mediated immune responses, including Th2 and Th17, play an important role in the inflammatory response of liver egg granuloma of schistosome infection [42]. In this study, we examined changes in cytokine levels associated with immune response in mice with anti-BAFF treatment. According to the results, anti-BAFF treatment could significantly reduce Th2 cytokine IL-4 and Th17 cytokine IL-17. IFN-γ levels associated with the Th1 response decrease, but there was no statistically significant difference. These results demonstrated that anti-BAFF treatment inhibited the development of the Th2 and Th17 responses induced by egg antigen and reduced the intensity of inflammatory response in egg granuloma.

IL-10 is usually produced by regulatory T cells, regulatory B cells and other B cells that secrete IL-10. IL-6 is usually produced by activated B effector cells [43,44]. IL-10-secreting Bregs are now regarded as negative regulators of the immune system, inflammation and autoimmunity, based on studies in humans and mouse models of autoimmune diseases such as rheumatoid arthritis, SLE and MS [42]. Effector B cells, which secrete IL-6, play an important role in inflammation [45]. IL-10 plays an important role in the inhibition of schistosomiasis liver fibrosis [10,46,47]. In this study, compared with the infected group, anti-BAFF treatment could increase the level of IL-10, decrease the level of IL-6, and decrease the level of specific antibodies against AWA and SEA, suggesting that anti-BAFF treatment may affect the maturation of the plasma cells that secrete antibodies. The maturation of plasma cells is related to the IL-6 secreted by B cells and follicle-assisted T cell function [45]. BAFF receptors exist on the surface of many cells and play an important role in the survival, differentiation and development of T and B. The results of this study suggest that anti-BAFF treatment may change the composition of T and B cell subsets, resulting in an increase in the number of regulatory T and B cell subsets secreting IL-10 and a decrease in the effector T and B cell subsets secreting IL-6, but this hypothesis requires further confirmation.

TGF-β plays a crucial role in liver fibrosis and is produced by both resident liver cells as well as infiltrating inflammatory cells, and this can activate fibroblasts, hepatic astrocytes (HSCs) to produce a large amount of the liver fibrotic matrix [48]. Connective tissue growth factor (CTGF), α-SMA, Col-IA2, differentiation inhibitor 1 (Id1), matrix metalloproteinase 2 (MMP-2) and integrin junction kinase (ILK) [49], as downstream targets of TGF-β, induced the transformation of fibroblasts into active myofibroblasts, secreting various extracellular matrix proteins [50]. In this study, the TGF-β level in the serum of mice in the anti-BAFF treatment group was significantly lower than that in the natural infection group, which may be an important reason for the significant reduction in the degree of liver fibrosis of mice in this group.

Interestingly, compared with mice that knocked out B cells [5,33], which worsened liver egg granuloma and fibrosis, the use of BAFF-neutralizing antibodies in this study alleviated the degree of granuloma and fibrosis in the liver of mice infected with schistosomiasis. The knockout of B cells in mice affected the function of B cells, thereby affecting the pathological changes in schistosomiasis-infected egg granulomas. BAFF is expressed in various cells and can affect the cellular functions related to schistosomiasis egg granuloma and fibrosis, such as T, B, monocytes, and hepatic stellate cells. In our study, anti-BAFF treatment could partially affect the function of B cells, downregulating Th2 and Th17 responses. Anti-BAFF treatment affected multiple cell functions, and its downregulation of schistosomiasis-infected egg granuloma and fibrotic lesions was the result of multiple cell functions being jointly affected, not just the result of B cell function being affected. It has been reported that the growth and development of schistosome is related to the host immune system [51,52,53]. Compared to B-cell-knockout mice infected for 5 weeks with a reduced egg load and returned to normal after 8 weeks [5], BAFF-neutralizing antibodies did not reduce egg loads in our study, and the possible reasons for this are similar to those presented above. The neutralization antibodies of BAFF partially damage the function of B cells, which may not be sufficient to affect adult reproductive ability and egg loads.

In summary, this study has shown that BAFF plays a significant role in the pathogenesis of schistosomiasis. Using anti-BAFF treatment, it down-regulated Th2 and Th17 responses, and B cell function, resulting in a reduction in the inflammatory reaction and fibrosis of the liver egg granuloma of the host infected with *S. japonicum*. The results of this study could help to further clarify the immune mechanism of hepatopathy in the host infected with schistosome and provide new ideas regarding the development of new methods against schistosomiasis hepatic pathogenesis.

## Data Availability

Not applicable.
